# Case Report: Emergency endovascular management of a ruptured giant abdominal aortic aneurysm with severely angulated and conical shaped neck using novel multiple stiff wire technique

**DOI:** 10.12688/f1000research.152300.1

**Published:** 2024-09-19

**Authors:** Taofan Taofan, Iwan Dakota, Suko Adiarto, Suci Indriani, Ruth Grace Aurora, Rendra Mahardhika Putra, Bagas Adhimurda Marsudi, Jonathan Edbert Afandy, Melani Limenco Benly, Kanya Paramastri, Macmilliac Lam, Muhammad Rafli Iqbal, Keviano Bobby Saputro, Sung Gwon Kang, Bambang Widyantoro

**Affiliations:** 1 Department of Cardiology and Vascular Medicine, Faculty of Medicine University of Indonesia, National Cardiovascular Center, Jakarta, Special Capital Region of Jakarta, 11420, Indonesia; 2 Department of Cardiology and Vascular Medicine, Faculty of Medicine, Universitas Airlangga, Dr Soetomo General Academic Hospital, Surabaya, Jawa Timur, 60268, Indonesia; 3 Research Assistant of Vascular Division, Department of Cardiology and Vascular Medicine, Faculty of Medicine University of Indonesia Academic Hospital, National Cardiovascular Center Harapan Kita, Jakarta, Special Capital Region of Jakarta, 11420, Indonesia; 4 Department of Radiology, Chosun University, Gwangju, Gwangju, South Korea

**Keywords:** Abdominal Aortic Aneurysm, Endovascular Aortic Repair, Multiple Stiff Wire Technique, Hostile Neck, Conical-Shaped Neck, Ruptured Giant AAA

## Abstract

**Background:**

Ruptured abdominal aortic aneurysm (rAAA) is commonly fatal, with an overall mortality rate of nearly 90%, and the risk of subsequent rupture remains high, especially in large aneurysm diameters or progressive disease. Unfavorable neck anatomy in EVAR is linked to early graft failure and long-term complications. Recently, a novel multiple stiff wire (MSW) technique has been developed to overcome the challenges of hostile neck anatomy without introducing additional devices and procedural complexity. It has also been feasible in a series of elective cases. In this case, we report the first-ever utilization of the MSW technique in an emergency case of an acute contained rAAA with a conical-shaped, severely angulated neck who underwent Endovascular Aortic Repair (EVAR).

**Case presentation:**

A 61-year-old man came with intermittent sharp stomach pain radiating to his back since three weeks ago. Physical examination showed elevated blood pressure and anemic conjunctiva. Laboratory examinations showed anemia, leukocytosis, elevated D-dimer level, high creatinine level, and low eGFR. CT-Scan Angiography (CTA) revealed severely hostile anatomy, a conical-shaped abdominal aorta aneurysm with a length of 13.2 cm and a maximum diameter of 9.3 cm with angulation of 90.1°. The patient was diagnosed with Ruptured AAA with a conical-shaped, severely angulated neck. Endovascular Aortic Repair (EVAR) management with MSW technique was planned for him. After four days, The patient was discharged in a clinically stable condition with optimal medical treatment and education.

**Conclusion:**

The endovascular approach could be performed in emergency settings and has been proven to reduce length of stay, mortality, and morbidity rates. In this case, the endovascular approach with the MSW technique showed promising results for the patient.

## Introduction

Abdominal Aortic Aneurysm (AAA) is increasing worldwide, ranging from 4.2% to 11% per year, as it is a degenerative process and almost exclusively occurs in the infrarenal region and can present as asymptomatic (majority of cases), symptomatic non-ruptured, and ruptured AAA (rAAA). When ruptured, AAA is commonly fatal, with an overall mortality rate of nearly 90%. ^1^ Rarely, the surrounding peritoneum can effectively seal off and contain the rupture, but the risk of subsequent rupture remains high especially in large aneurysm diameters or progressive disease. ^2^

Endovascular Aortic Repair (EVAR) remains the cornerstone of AAA management and is generally favored over open surgical repair. However, EVAR presents many challenges, one of which is limited due to challenging anatomy. A hostile neck remains one of the most common reasons a vascular specialist might not recommend EVAR. ^3^ Studies have found unfavorable neck anatomy to be linked to early graft failure and long-term complications such as stent graft collapse, migration, type I endoleak, and late aneurysm rupture. ^4^

Recently, a novel multiple stiff wire (MSW) technique has been developed to overcome the challenges of hostile neck anatomy without introducing additional devices and procedural complexity. It has also been feasible in a series of elective cases. ^5^ In this case, we report the first-ever utilization of the MSW technique in an emergency case of an acute contained rAAA with a conical-shaped, severely angulated neck who underwent EVAR.

## Case report

A 61-year-old man came with intermittent sharp stomach pain that was referred to his back for three weeks. The patient had no prior history of abdominal, flank, or lower back pain before his presentation at our hospital. Other complaints, such as chest pain, shortness of breath, coughing, fever, and bloody stool, were denied.

**Figure 1.**
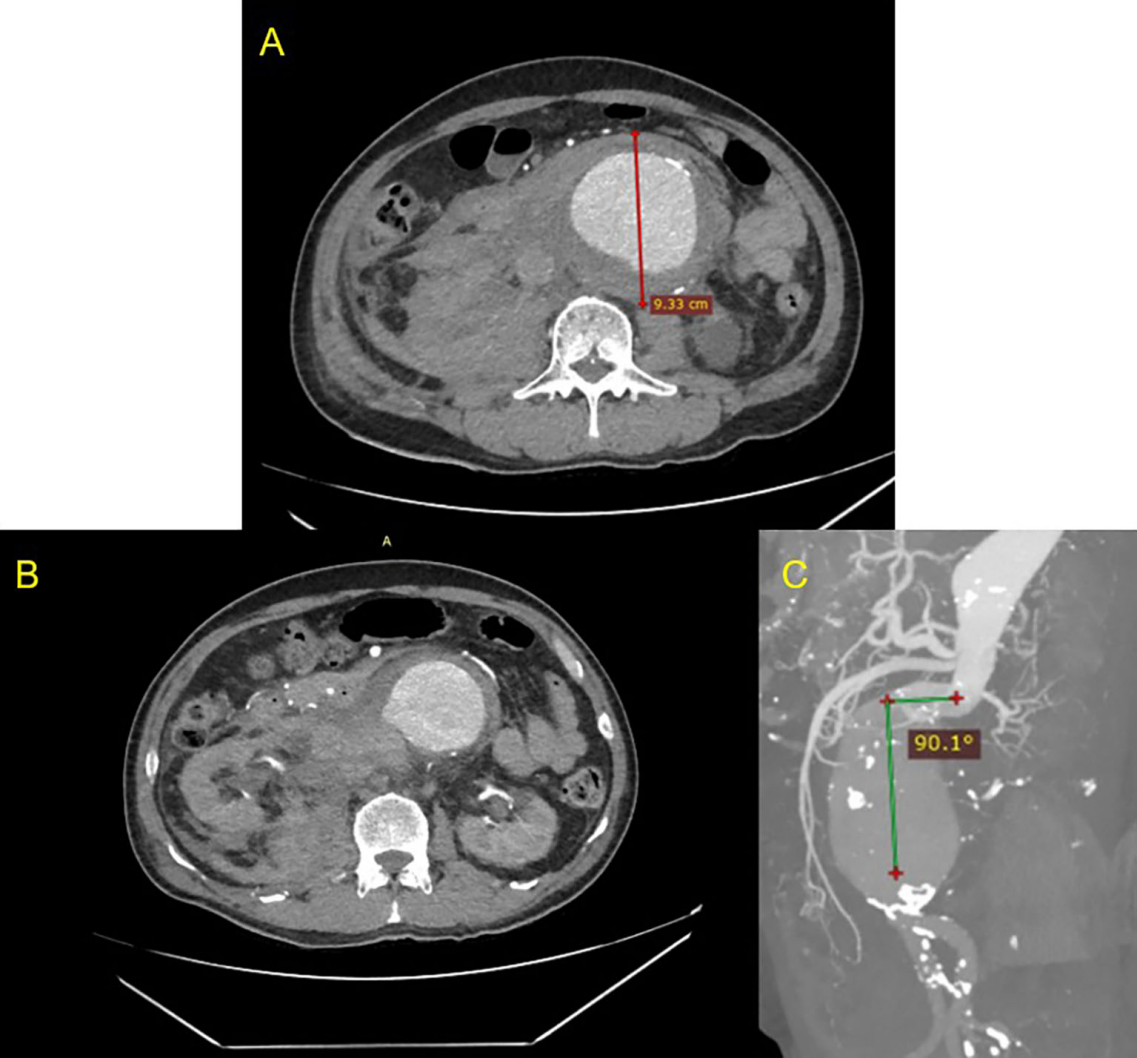
CT Scan of the patient before intervention. A. Pre-procedural axial slice CT Scan angiography showing giant abdominal aortic aneurysm; B. Axial CT Scan angiography showing ruptured aneurysm; C. Maximum Intensity Projection of CT Scan showing anatomy features of the conical neck and severe angulation (90.1°)

Physical examination showed elevated blood pressure (148/90 mmHg), regular heart rate (73 bpm), elevated respiratory rate (22×/min), and normal oxygen saturation (98%). Anemic conjunctiva was found, although other physical examinations appeared within normal range. Laboratory examinations showed anemia (Hb 7.7 g/dL), leukocytosis (10.920 cells/μL), elevated D-dimer level (12.410 μg/mL), high creatinine level (2.01 mg/dL), and low eGFR (37 mg/mL/min/1.73 m ^2^). CT-Scan Angiography (CTA) revealed a ruptured ( Figure 1B) and conical-shaped abdominal aorta aneurysm with a length of 13.2 cm, maximum diameter of 9.3 cm ( Figure 1A), and 90.1° angulation ( Figure 1C).

The patient was diagnosed with Ruptured AAA with a conical-shaped, severely angulated neck, alongside anemia due to gastrointestinal bleeding, controlled hypertension, and CKD. EVAR management with multiple stiff wire techniques was planned for him.

**Figure 2.**
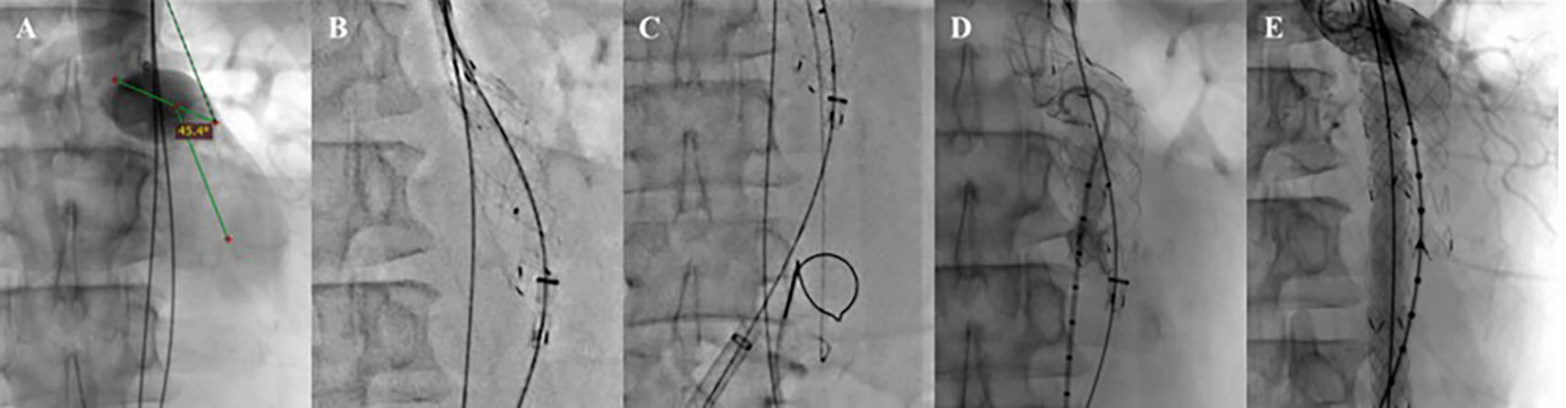
EVAR Procedure. A. Proximal Neck Angulation; B. Main Body Deployment; C. Wiring Attempt; D. Aortography; E. Final Aortography.

The EVAR procedure was done using the MSW technique. ^5^ After general anesthesia, vascular access to the right femoral artery (RFA) and left femoral artery (LFA) was established, followed by the insertion of a guidewire with a support catheter through both femoral arteries, which was then positioned in the descending thoracic aorta. Wire exchanges with 0.035″ × 260 cm Extra Stiff Wire (Cook, Bloomington, USA) were done in both accesses. Initial aortography from right brachial artery access revealed a contained rupture of the infrarenal aorta. The proximal neck angulation was decreased to 45.4° ( Figure 2A). A SEAL Novus Main Body Stent Graft ^®^ sized 26 mm × 50 mm main body (S&G Biotech, Yongin, Korea) was deployed via the RFA ( Figure 2B). Then, a wiring attempt for intragraft cannulation via RFA was done but failed, so it was decided to do wire snaring from brachial artery access to the LFA access ( Figure 2C). Aortography with a pigtail catheter via LFA was done to confirm the wire position ( Figure 2D). Wire exchange with 0.035″ × 260 cm Extra Stiff Wire (Cook, Bloomington, USA) was done, and SEAL Novus Limb Stent Graft ^®^ sized 12 mm × 80 mm (S&G Biotech, Yongin, Korea) was deployed from abdominal aorta until left common iliac artery. Another SEAL Novus Flared Limb Stent Graft ^®^ 12 mm × 100 mm (S&G Biotech, Yongin, Korea) was deployed until the left external iliac artery overlapped with the previous stent. For the right common iliac artery, SEAL Novus Limb Stent Graft ^®^ 12 mm × 120 mm (S&G Biotech, Yongin, Korea) was deployed, overlapping with the previous stent. Final aortography revealed an excellent position of the stent grafts. The contrast filled the whole cover stent without a sign of endoleak ( Figure 2E). A 270 mL Iodixanol 370 mg/mL contrast was used, dose area product 180.68 Gy.cm ^2^, and fluoroscopy time was 1 hour 05.54 minutes.

**Figure 3.**
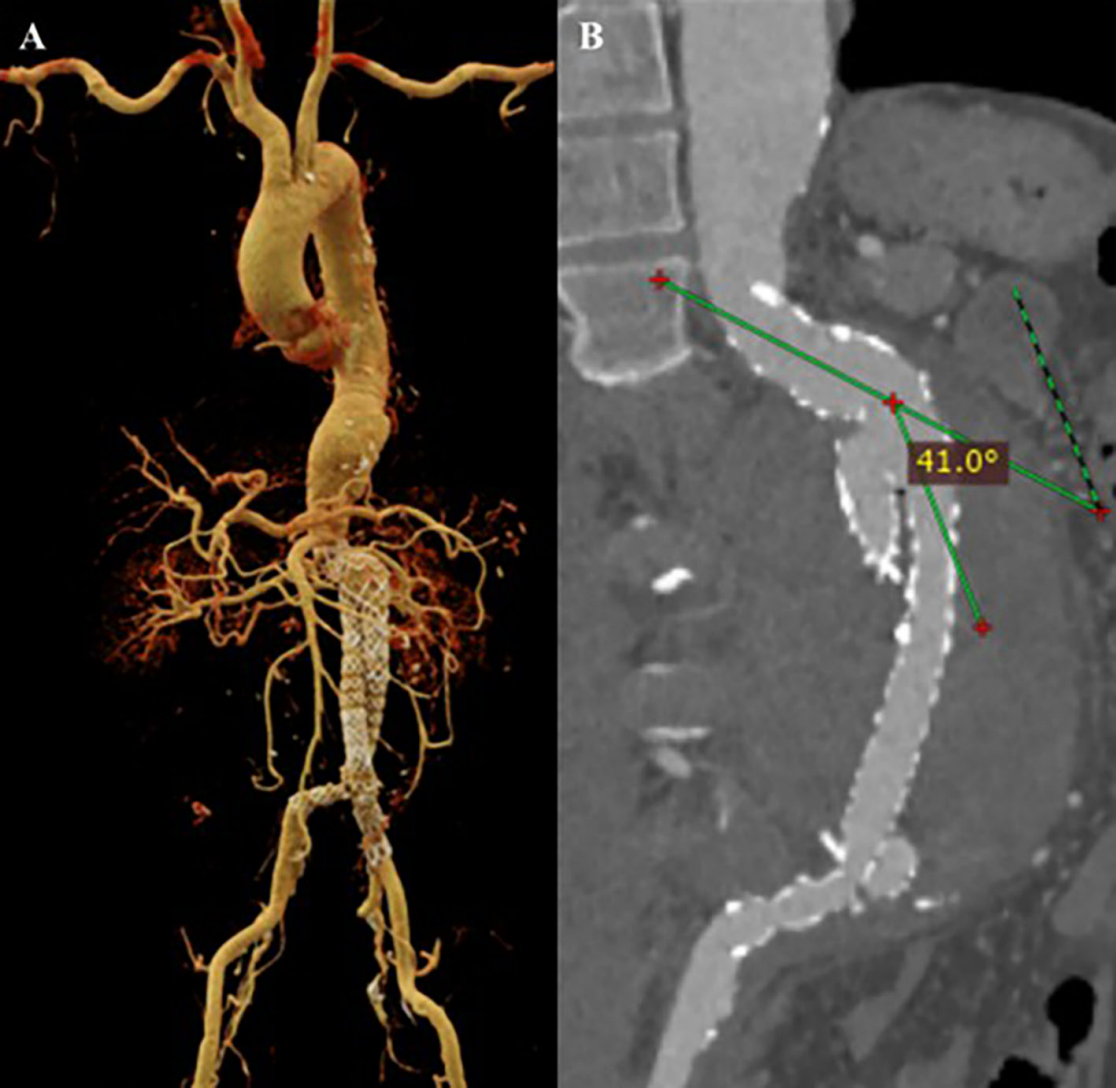
CT Scan after EVAR procedure. A. volume rendering shows the stent graft position with no evidence of endoleak; B. Maximum Intensity Projection of CT scan shows anatomy features of the conical neck and angulation of the abdominal aorta.

After the procedure, the patient was observed in the intensive cardiovascular care unit. Upon laboratory testing, the patient’s hemoglobin went up to 11 mg/dL. The evaluation CTA indicated an optimal stent location without migration, no endoleak was detected, and a decreased proximal neck angulation of 41.0° ( Figure 3). After four days, the patient was discharged in a clinically stable condition with optimal medical treatment and education. The patient was prescribed spironolactone, bisoprolol, candesartan, rosuvastatin, sucralfate, lansoprazole, and ibuprofen as pharmacotherapy.

## Discussion

The emergency EVAR approach for rAAA was initially implemented in 2004, with published findings indicating a reduction in the 30-day postoperative death rate (17.9% vs. 30.0%). ^6^ Compared to open surgery, a US database cohort found in-hospital mortality was significantly lower for EVAR (23.8% vs. 36.3%), and similar results were found after analysis of the Vascular Quality Initiative database (23% vs. 35% for EVAR vs. open repair). ^7^^,^^8^ Endovascular repair provides a superior option, particularly in patients of advanced age with chronic pulmonary disease, cardiovascular disease, and renal dysfunction. It is associated with a threefold reduction in perioperative mortality, even in younger patients with fewer comorbidities. ^9^

The clinical profile of this patient was all in favor of the emergency EVAR procedure. The patient had old age, cardiovascular disease, and renal dysfunction, which were determinants of periprocedural mortality and morbidity, as well as worse outcomes if open surgical repair was attempted.

In our case, the momentous challenge of the hostile neck anatomy with severe neck angulation remains. Endovascular aneurysm repair (EVAR) with pronounced neck angulation, in contrast to mild neck angulation, exhibited a markedly elevated incidence of type 1a endoleak over three years (5.6% versus 2.6%), neck-related secondary interventions over three years (13.1% versus 9%), migration rates within one year (5.4% versus 4.0%), and both aneurysm-related and all-cause mortality within one year (6.4% versus 4.3%). ^10^

Several techniques have been proposed to overcome the anatomical challenges of severe neck angulation. The Kilt technique was recently developed to modify AAA neck angulation and proved effective with 100% technical success and without any development of endoleaks during long-term follow-up. ^11^ Our cohort developed the MSW technique and involved a similar method, introducing additional wires to further control and modify the neck angle. The MSW technique demonstrated promising results in the number of thoracic and abdominal endovascular repairs. It enables neck angulation modification of the aortic neck without added procedure complexity, the need for specialized devices, or special training for the operator. It also comes with the benefit of reduced cost compared to specialized devices. ^5^ The MSW technique is the perfect solution for emergency cases in developing countries where specialized devices are limited. With the MSW technique, there’s no need for expensive equipment, which makes it an affordable and practical choice.

This case report represents the first-ever application of the MSW technique in an emergency EVAR protocol in a contained ruptured giant AAA with a hostile neck. The patient was at high intraoperative risk because of the severe angulation and conical-shaped neck anatomy.

## Conclusion

Our case illustrates the successful application of the MSW technique in an emergency EVAR for a challenging anatomical scenario. This approach highlights the potential of innovative methods in improving outcomes for patients with ruptured AAAs, underscoring the importance of continued advancements in endovascular interventions.

## Consent

Informed consent has been obtained from the patient to publish the case report and accompanying images.

## Data Availability

No data are associated with this article. Figshare: CARE checklist for “Emergency endovascular management of a ruptured giant abdominal aortic aneurysm with severely angulated and conical shaped neck using novel multiple stiff wire technique”. https://doi.org/10.6084/M9.FIGSHARE.26976100.V1. ^12^ Data are available under the terms of the Creative Commons Attribution 4.0 International license (CC-BY 4.0).
