# Supplementary material for: Case Report: Emergency endovascular management of a ruptured giant abdominal aortic aneurysm with severely angulated and conical shaped neck using novel multiple stiff wire technique
Source: F1000Res. 2025 Feb 19;13:1074. Originally published 2024 Sep 19. [Version 2] doi: 10.12688/f1000research.152300.2 (PMC12000802; doi:10.12688/f1000research.152300.2)
Supplement: Peer review history

# Reviewer report: Reviewer response for version 2

**DOI:** 10.5256/f1000research.178367.r372633

**Published:** 2025-04-15

**Authors:** Karthigesu Aimanan

**Affiliations:** 1 Hospital Kuala Lumpur, Kuala Lumpur, Malaysia

1. **Clarification Required:** In the sentence:

*“A SEAL Novus Main Body Stent Graft® sized 26 mm × 50 mm (S&G Biotech, Yongin, Korea) was deployed via the RFA (Figure 2B). Then, a wiring attempt for intragraft cannulation via RFA was done but failed, so it was decided to do wire snaring from brachial artery access to the LFA access (Figure 2C).”*

 There appears to be an error. The intragraft cannulation attempt should logically be from the **LFA**, not the **RFA**, as the contralateral limb gate is typically cannulated from the left side. Please revise this for accuracy.

 2. **Ballerina Configuration:** Kindly elaborate whether the ballerina configuration seen in the final 3D reconstruction was **intentional or accidental**. Although not mentioned in the operative steps, the final imaging suggests a ballerina configuration. Clarifying this would enhance the understanding of the procedural outcome.

 3. **Kilt Technique:** Since the **Kilt technique** is referenced in the discussion, a brief elaboration would be beneficial.

 4.Please share some evidence of much neck angulation will be corrected by this multi wire technique based on literatures is there any.

Are enough details provided of any physical examination and diagnostic tests, treatment given and outcomes?

Yes

Is the case presented with sufficient detail to be useful for other practitioners?

Yes

Is sufficient discussion included of the importance of the findings and their relevance to future understanding of disease processes, diagnosis or treatment?

Yes

Is the background of the case’s history and progression described in sufficient detail?

Yes

Reviewer Expertise:

vascular and endovascular surgery

I confirm that I have read this submission and believe that I have an appropriate level of expertise to confirm that it is of an acceptable scientific standard.

# Reviewer report: Reviewer response for version 1

**DOI:** 10.5256/f1000research.167041.r329304

**Published:** 2024-10-31

**Authors:** Muhamad Taufik Ismail

**Affiliations:** 1 Sardjito Central General Hospital/Faculty of Medicine, Public Health, and Nursing, Gadjah Mada University,, Yogyakarta, Indonesia

I have reviewed the case report titled *"Emergency Endovascular Management of a Ruptured Giant Abdominal Aortic Aneurysm with Severely Angulated and Conical-Shaped Neck Using a Novel Multiple Stiff Wire Technique"* and have identified several areas for improvement:

1. **Background and Introduction**: The background effectively emphasizes the significance of ruptured abdominal aortic aneurysms (rAAA) and the technical challenges associated with endovascular aneurysm repair (EVAR) in anatomically complex cases, such as those with severely angulated necks. The inclusion of information about abdominal aortic aneurysms, their prevalence, and associated healthcare burdens provides a strong foundation for discussing this case. However, a specific description of the multiple stiff wire (MSW) technique and the rationale for selecting this technique over other alternatives is missing. Please include a brief overview of other commonly used techniques in the introduction, especially those applicable to EVAR in cases with hostile neck anatomy, like the triple wire technique, which also does not require additional devices.
2. **Technical Details and Illustrative Aids**: Given that the multiple stiff wire technique is relatively novel, it would enhance reader comprehension to provide a more comprehensive explanation of this method in the case section. Consider including a detailed step-by-step description of the procedure or summarizing it with images to aid visualization. Additionally, an explanation of its advantages and disadvantages would be valuable.
3. **Terminology**: Instead of using “first ever utilization,” consider a more nuanced phrasing such as, *“To our knowledge, there are no reported or published articles regarding the use of the multiple stiff wire technique in the emergency management of ruptured abdominal aortic aneurysm.”* This wording preserves the novelty of the approach while avoiding overly definitive claims.
4. **Post-Procedure Outcomes**: The post-procedure assessment should include a detailed evaluation of potential adverse events, especially the impact on renal function, as this patient had an initially abnormal renal function test, which may be affected by contrast administration. Additionally, consider including other clinical outcomes, such as cardiovascular or cerebrovascular complications, and provide longer follow-up data where possible. A follow-up CTA at 1 month (or longer if available) is recommended to assess complications such as endoleak, possible shortening or migration etc.

Are enough details provided of any physical examination and diagnostic tests, treatment given and outcomes?

Yes

Is the case presented with sufficient detail to be useful for other practitioners?

Partly

Is sufficient discussion included of the importance of the findings and their relevance to future understanding of disease processes, diagnosis or treatment?

Yes

Is the background of the case’s history and progression described in sufficient detail?

Partly

Reviewer Expertise:

vascular medicine, peripheral intervention, coronary intervention, neovascularization, nanomedicine,  molecular, regenerative,  and precision medicine

I confirm that I have read this submission and believe that I have an appropriate level of expertise to confirm that it is of an acceptable scientific standard.
